# Fetal Presentation of Mediastinal Immature Teratoma: Ultrasound, Autopsy and Cytogenetic Findings

**DOI:** 10.3390/diagnostics11091543

**Published:** 2021-08-25

**Authors:** Maria Paola Bonasoni, Giuseppina Comitini, Veronica Barbieri, Andrea Palicelli, Nunzio Salfi, Gianluigi Pilu

**Affiliations:** 1 Pathology Unit, Azienda Unità Sanitaria Locale—IRCCS di Reggio Emilia, 42122 Reggio Emilia, Italy; Andrea.Palicelli@ausl.re.it; 2 Department of Obstetrics & Gynaecology, Azienda Unità Sanitaria Locale—IRCCS di Reggio Emilia, 42122 Reggio Emilia, Italy; Giuseppina.Comitini@ausl.re.it; 3 Medical Genetics, Laboratory, Azienda Unità Sanitaria Locale—IRCCS di Reggio Emilia, 42122 Reggio Emilia, Italy; Veronica.Barbieri@ausl.re.it; 4 Pathology Unit, IRCCS Istituto Giannina Gaslini, 16147 Genoa, Italy; nunziosalfi@gaslini.org; 5 Obstetric Unit, Department of Medical and Surgical Sciences, Sant’Orsola-Malpighi Hospital, 40138 Bologna, Italy

**Keywords:** second trimester ultrasound, immature teratoma, karyotype

## Abstract

Teratomas are the most common congenital tumors, occurring along the midline or paraxial sites, or uncommonly, the mediastinum. Teratomas are classified as mature, containing only differentiated tissues from the three germinal layers; and immature, which also present with neuroectodermal elements, ependymal rosettes, and immature mesenchyme. Herein, we describe a new case of fetal mediastinal immature teratoma detected at 21 weeks of gestational age (wga) + 1 day with thorough cytogenetic analysis. Ultrasound (US) showed a solid and cystic mass located in the anterior mediastinum, measuring 1.8 × 1.3 cm with no signs of hydrops. At 22 wga, US showed a mass of 2.4 cm in diameter and moderate pericardial effusions. Although the prenatal risks and available therapeutic strategies were explained to the parents, they opted for termination of pregnancy. Histology showed an immature teratoma, Norris grade 2. Karyotype on the fetus and tumor exhibited a chromosomal asset of 46,XX. The fetal outcome in the case of mediastinal teratoma relies on the development of hydrops due to mass compression of vessels and heart failure. Prenatal US diagnosis and close fetal monitoring are paramount in planning adequate treatment, such as in utero surgery, ex utero intrapartum therapy (EXIT) procedure, and surgical excision after birth.

## 1. Introduction

Teratomas are the most common congenital tumours, representing 16.6% of all fetal neoplasms with an incidence of 1:20,000 to 1:40,000 livebirths [1,2,3]. They usually arise in a para-axial or midline location, including the brain, sacrum, and gonads. The sacroccyx is the main location (40%), and the mediastinum accounts for only 4% of cases [4,5].

Fetal mediastinal teratomas are rare and usually arise in the anterior mediastinum as a median or paramedian mass [6]. At prenatal ultrasound (US), they appear multilobulated with solid and cystic areas, sometimes with calcifications, and acoustic shadows [7,8]. The differential diagnosis includes bronchogenic cysts, congenital cystic adenomatoid malformation (CCAM) of the lung, diaphragmatic hernia, or bronchopulmonary sequestration [4,9]. Although located in the mediastinum, fetal intrapericardial teratomas are considered cardiac tumors, as their origin is typically from the right anterior border of the heart [10,11].

Fetal mediastinal teratomas are a type of Extragonadal Germ Cell Tumour (EGCT) and differentiate in tissues from the three germinal layers (ectoderm, mesoderm, endoderm). They are divided into mature and immature, the former being the majority. Immature teratomas are uncommon, representing only 1%. They are made up of neuroectodermal epithelium with neuroglial elements and ependymal rosettes; immature mesenchyme may also be present [12].

The thoracic location may cause compression of lungs, heart, and major vessels, resulting in pericardial and pleural effusions, pulmonary hypoplasia, and cardiac failure. The main clinical consequences are non-immune fetal hydrops (NIFH) and polyhydramnios, but severe fetal distress and even stillbirth may occur [7,9].

Second trimester US detection of mediastinal teratomas has been scarcely reported [2,7,8,13,14], including the immature type [2,7,13,14].

Herein, we present a new case of fetal mediastinal immature teratoma first observed at US in the second trimester, at 21 weeks of gestational age (wga) + 1 day, initially with no signs of hydrops. Immature teratoma was then confirmed at autopsy and histology. Karyotyping was carried out on both the fetus and the neoplasia. Both showed a normal female chromosomal asset of 46,XX. To the best of our knowledge, this is the first fetal case in which cytogenetic analysis on the tumor was performed.

## 2. Case Description

A 38 year-old primigravida underwent a routine US scan at 21 wga+ 1 day, which showed a suspicious anterior mediastinal mass of 1.8 × 1 cm. The mother was immediately referred to an expert sonographer (Professor G.P.). Previous scans (at 12 and 18 wga), pregnancy and mother’s health were otherwise unremarkable.

**Figure 1:**
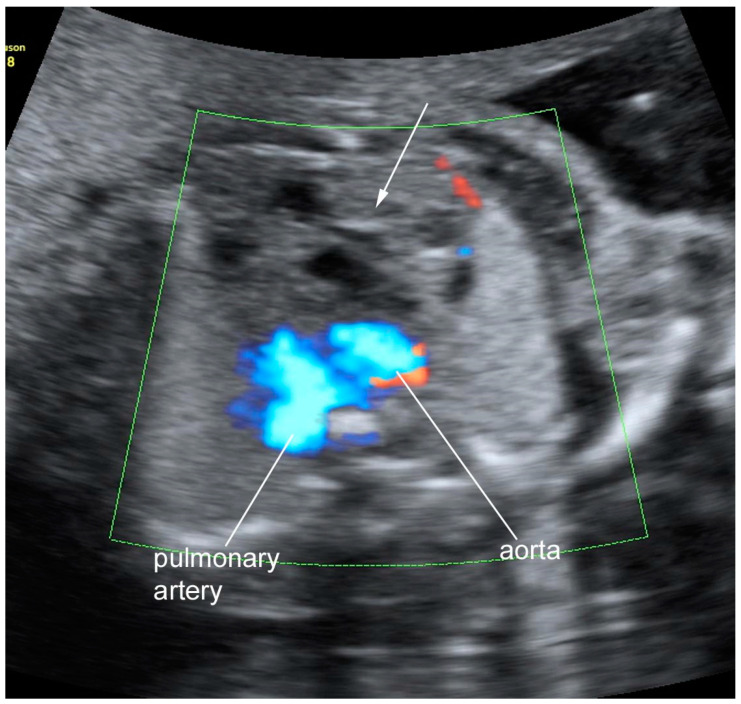
Ultrasound image of the teratoma: transverse view of the fetal chest at 22 weeks; a complex mass with solid and liquid components was seen in the upper and anterior part of the thorax (arrow).

Detailed US examination at 22 wga revealed a solid rounded echogenic mass in the anterior mediastinum with a minimal cystic component measuring 2.4 cm in maximum diameter. The mass dislocated inferiorly the main great vessels (aorta and pulmonary artery), posteriorly the aortic arch, laterally the superior vena cava, and superiorly the innominate vein (Figure 1). The heart showed normal anatomy with regular systemic and pulmonary venous return, atrio-ventricular and ventricular-arterial connections. Cardiac function, heartbeats, and rhythm were normal. A moderate pericardial effusion was present, but the pleural cavities were free of fluid. No fetal hydrops was present. The thymus was not clearly assessed. No other anomalies were observed at US.

The parents were made aware of the most probable diagnosis due to the anterior thoracic location (fetal mediastinal teratoma) and were explained the potential risks during pregnancy such as NIFH and polyhydramnios. All the possible therapeutic strategies potentially performed both during pregnancy and at birth were also illustrated, such as amniotic fluid reduction, ex utero intrapartum therapy (EXIT) or neonatal surgery in an adequate hospital structure. Moreover, the favorable neonatal outcome after surgical removal of mediastinal masses was also explained. Nevertheless, the parents opted for legal termination of pregnancy at 22 wga + 2 days. During the procedure, amniotic fluid was collected for karyotyping, which showed a normal female 46,XX formula.

Autopsy showed a female fetus with no significant dysmorphic features. Fetal anthropometric parameters and organ weights were assessed according to Archie JG et al. [15].

**Figure 2:**
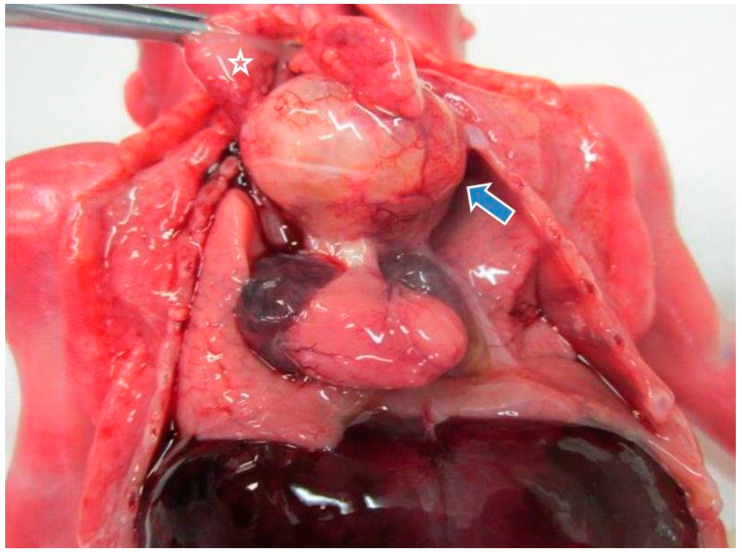
Gross features of mediastinal teratoma (blue arrow) at autopsy: the tumor was located underneath the thymus (star) and above the heart.

**Figure 3:**
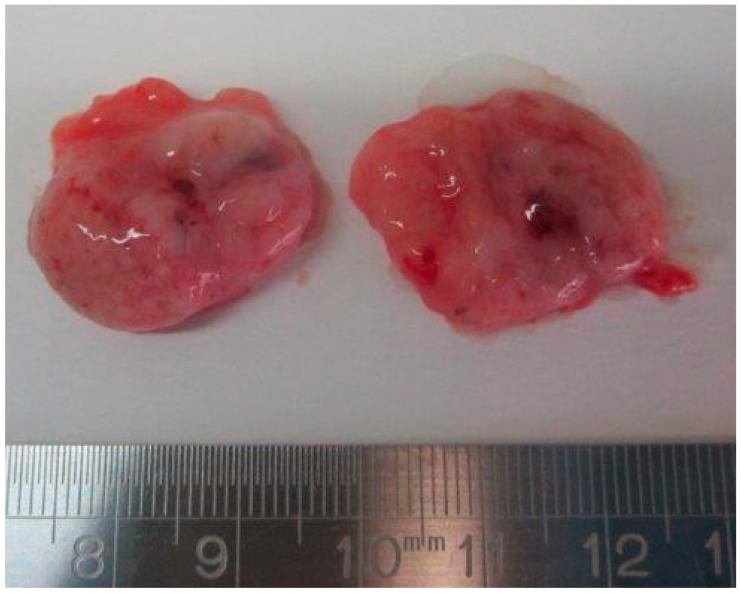
Dissected appearance of the teratoma: a pinkish solid, but soft nodule with cystic areas.

Internal examination revealed a pinkish nodule of 2.5 × 1.5 × 0.8 cm located in the anterior mediastinum underneath the thymus and above the pericardium (Figure 2). The nodule was easily dissected both from the posterior thymic surface and the anterior pericardium. On dissection, the nodule was mainly made up of solid pinkish tissue with focal cystic areas (Figure 3). Pericardial effusion was moderate with 0.5 mL of serous fluid. The combined lung weight was 8.7 g. Although not indicative of hypoplasia, the weight was in the lower range of what is expected for age (12.0 ± 3.8 g) [15]. Grossly, no other anomalies were identified.

**Figure 4:**
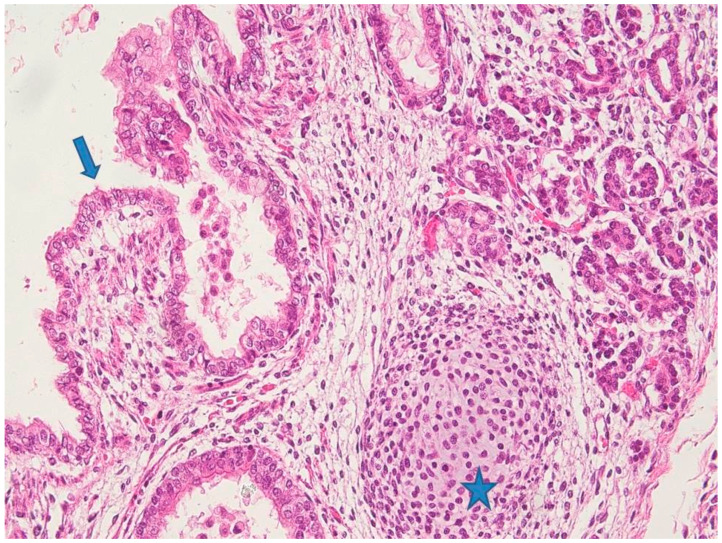
Histology of mature components of the neoplasia: endodermal elements with respiratory cilitaed epithelium (blue arrow) and glands (top right of the picture). Cartilage (mesoderm) was also present (star). (Hematoxylin Eosin (HE) staining 10×).

**Figure 5:**
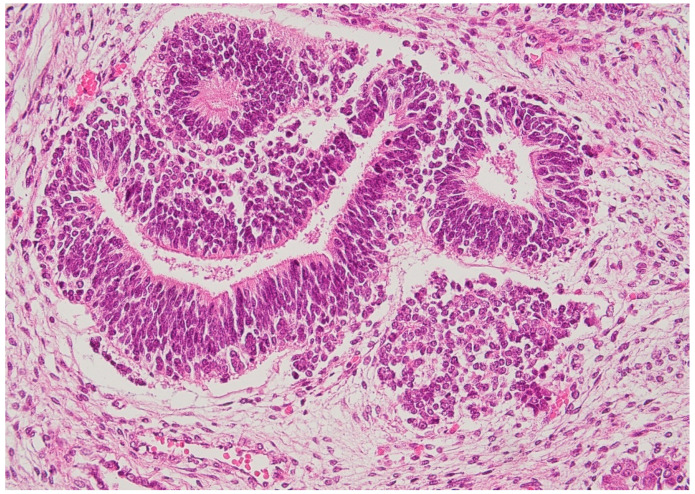
Immature elements of the tumor: neuroectodermal structures with ependymal rosettes (HE staining 20×).

**Figure 6:**
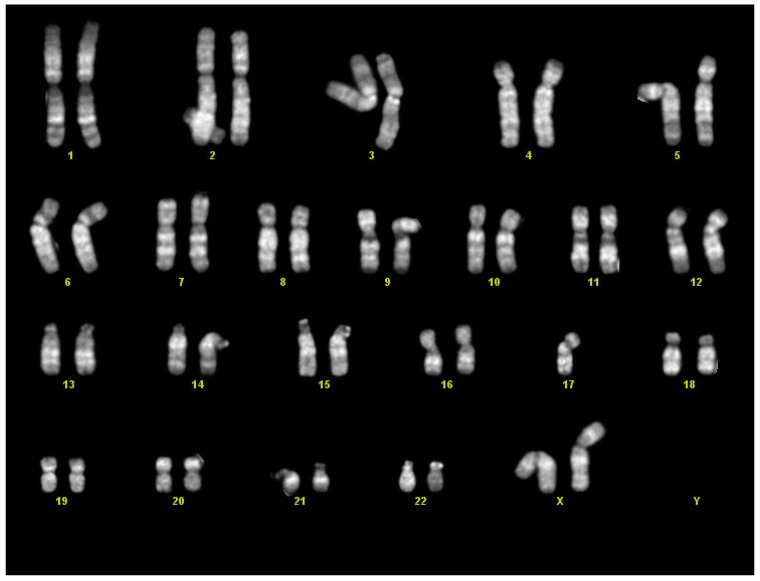
Tumor karyotype: female asset of 46,XX.

Microscopically, the nodule showed the typical features of a teratoma, composed of mature and immature tissue from the three embryonic germinal layers. Endodermal elements were predominant with ciliated respiratory epithelium, gastric mucosa, and glands in a lobular pattern (Figure 4). A miniature fetal liver (endoderm origin) with hematopoiesis (mesoderm) was focally identified. The mesodermal component was represented by choroid pigmented epithelium, renal structures, cartilage, abundant areas of immature mesenchyme, and smooth muscle. Neuroectoderm was abundant and characterized by immature neuroepithelium with ependymal rosettes (Figure 5). Although not validated for fetal teratomas, the application of Norris grading system would classify the neoplasia as immature grade 2 [16,17]. Cytogenetic analysis was carried out on the neoplasia, which showed 46,XX, a chromosomal asset identical to the somatic counterpart (Figure 6).

## 3. Discussion

Prenatal US diagnosis of mediastinal teratoma has been scarcely reported, especially in the second trimester, and is summarized in Table 1 [2,7,8,9,13,14,18,19,20,21,22,23,24].

In our case, a mediastinal mass was detected at US at 21 wga + 1 day, being the second fetal case reported both with an early diagnosis and without hydrops. Only one case, at 21 wga + 6 days has been described without hydrops [2]. Instead, the other fetus, diagnosed at 20 wga, showed NIFH [14].

Overall, in the literature data retrieved, NIFH was a frequent finding, ranging from mild signs (pleural or pericardial effusions) to more severe features (skin edema, pulmonary hypoplasia, ascites, polyhydramnios). Therefore, prenatal US diagnosis is fundamental in identifying the type of mediastinal mass, early signs of NIFH and further therapeutic planning.

In fact, at US, mediastinal teratoma is typically characterized by progressive mass growth, and CCAM or pulmonary sequestration remain unchanged or even decrease in size at consecutive scans [2,25,26]. In our case, the teratoma was rapidly growing in a short time. US scans at 12 and 18 weeks were normal, but at 21 + 1 wga, the mass measured 1.8 × 1.3 cm with no accompanying hydrops. At 22 weeks, the neoplasm reached 2.4 cm in maximum diameter and a mild to moderate pericardial effusion was detected with major vessels displacement. At autopsy, carried out at 22 + 2 wga, the dimensions recorded were 2.5 × 1.5 × 0.8 cm.

In our case, histopathological examination revealed an immature teratoma, Norris grade 2. The immature type seems the most common in the prenatal setting [2,4,7,13,14,18,19], including three cases diagnosed only at birth with normal prenatal US [27,28,29]. In other fetal presentations of mediastinal teratoma, diagnosed only at autopsy, tumor differentiation was not mentioned [30,31].

In three cases [31], teratoma was found at autopsy with other associated anomalies, but karyotype was not performed. However, in most fetuses or newborns, teratoma was an isolated finding with no other associated anomalies, and the somatic karyotype was normal [13,18,22] or even not investigated [2,4,7,8,9,14,19,20,21,23,24,27,28,29,30,31]. None had karyotyping carried out on the tumor. Nonetheless, mediastinal teratoma may occur in Klinefelter syndrome and isochromosome 12p is considered indicative of malignant potential [32,33]. In our case, teratoma was an isolated finding with no other associated anomalies. Cytogenetic analysis displayed a normal karyotype of 46,XX in both the fetus and the neoplasia. These findings correlate with the current literature, as in the pediatric population, the tumor karyotype is normal independently of its grade of maturation and differentiation [34,35]. Moreover, as reported in children, mediastinal teratomas are reported more frequently in females [5,36,37]. To the best of our knowledge, our case was the first fetus in which a thorough cytogenetic analysis was performed.

In general, in newborns, after surgical removal, mature and immature mediastinal teratomas have a favorable prognosis with low mortality and good health at different follow up durations (Table 1) [38,39,40,41,42,43]. Although infrequent, immature teratomas may recur and a close clinical follow-up including tumor markers is necessary [44,45]. On the other hand, in the prenatal setting, fetal outcome of mediastinal teratomas relies on the development of NIFH, due to tumor size and progressive compression of venous return. Prenatal treatments include fetal aspiration of the cystic mass, amnioreduction in the case of polyhydramnios, and, where available, fetal surgery to avoid NIFH or its worsening. Timing and decision of postnatal excision depends on fetal conditions and mass size, and it should be organized in adequate hospital structures. The optimal approach is the ex utero intrapartum therapy (EXIT)-to-resection procedure, which allows immediate fetal thoracotomy and surgical removal of the mass, preventing acute respiratory distress [2].

In general, although the literature data is scarce, the prognosis of fetal mediastinal teratomas has been reported as favorable overall, if promptly managed during pregnancy, and it is closely connected to NIFH occurrence. In the case we presented, fetal outcome was uncertain as the mass was rapidly growing and worsening of NIFH was highly likely or even inevitable. Moreover, as the immature type is considered potentially malignant with a high risk of recurrence, this histological finding was another adverse prognostic factor. No data is available regarding the karyotype in fetal teratoma and its predictive value. Therefore, cytogenetic analyses should be helpful in exploring this unknown prognostic factor in the fetal/perinatal setting.

In conclusion, in our specific case, although it is only speculative, fetal prognosis would have probably been negative, as tumor growth was rapidly progressive and NIFH evolution may have resulted in fetal death.

## Figures and Tables

**Table 1:** Fetal cases currently reported with prenatal ultrasound (US) of mediastinal teratoma. Wga: weeks of gestational age; NIFH: Non Immune Fetal Hydrops; Y: yes; NOS: not otherwise specified. TOP: termination of pregnancy.

Author and Year	Wga at US Diagnosis	Teratoma US Findings	NIFH	Teratoma Histological Diagnosis	Outcome	Surgery
Weinraub et al. [9] 1989	29	Multilocular and cystic	Y: diffuse fetal edema, pleural effusions; pulmonary hypoplasia, placental hydrops and polyhydramnios	Mature	Fetal demise at 29 weeks	No
Dumbell et al. [20] 1990	35	Multilocular and cystic	Y: skin edema, polyhydramnios	Mature	Induction of labour; vaginal delivery at 36 weeks, baby alive at 18-month- follow-up	Y: 2 days after birth
Froberg et al. [4] 1994	25	Solid and cystic	Y: fetal anasarca	Immature	IUFD at 27 weeks: induction of labour, vaginal delivery	
Liang et al. [21] 1998	38	Solid and cystic	No: polyhydramnios	Mature	Delivery at 39 weeks	Y: 7 days after birth
Schild et al. [22] 1998	27	Solid	Y: skin edema, pleural effusions, ascites	Mature	C-section at 27 weeks + 5 days: failed resuscitation	
Wang et al. [18] 2000	36	Multilocular cystic	No; polyhydramnios at 38 weeks	Immature	Induction of labor, vaginal delivery at 39 weeks; alive after 3 months after surgery	Y: 4 days after birth
Aksoy et al. [23] 2002	34	Solid and cystic	Y: edema of head and neck, polyhydramnios, pulmonary hypoplasia	Immature	C-section at term; died at one day of life	Y: immediately after birth
Merchant et al. [7] 2005 Case 1	21 weeks + 4 days	Heterogeneous mass with calcifications	Y: diffuse edema, pleural effusions, ascites	Immature	C-section at 25 weeks for preterm labor; alive at 9-month-follow-up	Y: in utero at 23 weeks
Merchant et al. [7] 2005 Case 2	34	Heterogeneous mass with calcifications	Y: chest wall, facial, and scalp edema, hepatomegaly, ascites, pleural effusions, and massive polyhydramnios	Immature	EXIT procedure, alive at 1 year follow-up	Y: during EXIT procedure
Takayasu et al. [8] 2010	23	Cystic mass	Y: developed at 29 weeks	Mature	Vaginal delivery at 39 weeks; alive at 6-month-follow-up	Y: at 30 days of age
Giancotti et al. [24] 2011	29	Heterogeneous appearance with calcifications	Y: pleural effusions, ascites, polyhydramnios	Teratoma NOS	C-section 32 weeks; alive at 8-month-follow-up	Y: 1 day after birth
Gaetani et al. [2] 2016	21 weeks + 4 days	Cystic	No	Immature	C-section at 33 weeks	
Gong et al. [19] 2016	27	Heterogenous lobulated	Y: diffuse and polyhydramnios	Immature	TOP at 27 weeks	
Darouich et al. [13] 2020	23	Solid and cystic	Y: diffuse skin edema, ascites, and pleural effusions	Immature	TOP at 24 weeks	
Srisupundit et al. [14] 2020	20	Solid and cystic	Y: subcutaneous edema, moderate pleural effusion and ascites	Immature	TOP at 23 weeks	
Present case	21 weeks + 1 day	Solid with focal cystic areas	No	Immature teratoma	TOP at 22 weeks	

## Data Availability

The data presented in this study is available on request from the corresponding author.
